# A microarray study of gene and protein regulation in human and rat brain following middle cerebral artery occlusion

**DOI:** 10.1186/1471-2202-8-93

**Published:** 2007-11-12

**Authors:** Nick Mitsios, Mohamad Saka, Jerzy Krupinski, Roberta Pennucci, Coral Sanfeliu, Qiuyu Wang, Francisco Rubio, John Gaffney, Pat Kumar, Shant Kumar, Matthew Sullivan, Mark Slevin

**Affiliations:** 1 School of Biology, Chemistry and Health Science, John Dalton Building, Manchester Metropolitan University, Chester Street, Manchester, UK; 2 Department of Neurology, Stroke Unit, Hospital Universitari de Bellvitge (HUB), Fundacio IDIBELL, L' Hospitalet de Llobregat, Barcelona, Spain; 3 Departamento de Farmacologia i Toxicologia, Institut d' Investigacions Biomediques de Barcelona (IIBB), CSIC-IDIBAPS, Barcelona, Spain; 4 Department of Pathology, Medical School, University of Manchester and Christie Hospital, Manchester, UK

## Abstract

**Background:**

Altered gene expression is an important feature of ischemic cerebral injury and affects proteins of many functional classes. We have used microarrays to investigate the changes in gene expression at various times after middle cerebral artery occlusion in human and rat brain.

**Results:**

Our results demonstrated a significant difference in the number of genes affected and the time-course of expression between the two cases. The total number of deregulated genes in the rat was 335 versus 126 in the human, while, of 393 overlapping genes between the two array sets, 184 were changed only in the rat and 36 in the human with a total of 41 genes deregulated in both cases. Interestingly, the mean fold changes were much higher in the human. The expression of novel genes, including p21-activated kinase 1 (PAK1), matrix metalloproteinase 11 (MMP11) and integrase interactor 1, was further analyzed by RT-PCR, Western blotting and immunohistochemistry. Strong neuronal staining was seen for PAK1 and MMP11.

**Conclusion:**

Our findings confirmed previous studies reporting that gene expression screening can detect known and unknown transcriptional features of stroke and highlight the importance of research using human brain tissue in the search for novel therapeutic agents.

## Background

Ischaemic stroke results from obstruction of blood flow in a major cerebral vessel and leads to deregulation of genes whose expression promotes ischemic neuronal death and subsequent neurological dysfunction [1,2]. Under ischemic conditions, energy metabolism fails, and severe reduction in mRNA and protein synthesis occurs in the ischemic core region. The tissue surrounding this area (peri-infarcted region) is able to maintain some functions, such as ionic homeostasis and can be partially salvaged by blood recirculation [3,4].

The precise molecular mechanisms involved in ischemia-induced brain injury remain poorly understood. Limited knowledge of the molecular mechanisms involved in tissue regeneration has been gained from animal experiments using the middle cerebral artery occlusion (MCAO) model which replicates, in many aspects, the neuropathological changes following stroke in humans [5]. Although the contralateral side of the brain is not totally unaffected by ischemic damage, the collection of experimental and reference control tissue from the same animal is a better comparison than using sham-operated control in rats, while in human samples the only control tissue available is contralateral hemisphere. In addition, using contralateral tissue as a control and the direct comparison with stroke hemisphere provides the best model for validation as it removes inter-patient genetic variation and also minimises the differences in potential degradation between the target and reference mRNAs. This has been applied previously in both human [6,7] and animal [8-10] studies. Rao et al. in particular observed very few differences in gene expression between sham and contralateral cortex at 24 h of reperfusion following MCAO in the rat [9].

Analysis of ischemic brain tissue with techniques capable of studying multiple transcripts simultaneously can identify gene expression changes previously not known to be implicated in ischemic pathophysiology and may lead to development of new targets for stroke therapy [11]. DNA microarray technology has been used to investigate the expression of thousands of genes in a single hybridization experiment. Several experimental studies have examined alteration of gene expression in the postischemic rat brain using microarray technology [8-10,12-18], while blood genomic profiling in human stroke have been investigated in recent pilot studies [19,20] (Table 1). Critical comparison of gene expression profiles after stroke in humans with those in animal models may lead to a better understanding of the pathophysiology of brain ischaemia and allow an evaluation of the usefulness of animal models in stroke research.

**Table 1:** Previous studies employing microarray approaches to study stroke

	Soriano et al. 2000	Jin et al. 2001	Kim et al. 2002	Rao et al. 2002	Schmidt-Kastner et al. 2002	Tang et al. 2002	Roth et al. 2003	Kim et al. 2004	Lu et al. 2004	Moore et al. 2005	Ford et al. 2006	Tang et al. 2006	Vikman and Edvinsson 2006	Our data
Material used	Rat brain tissue	Rat brain tissue	Rat brain tissue	Rat brain tissue	Rat brain tissue	Rat brain tissue	Rat brain tissue	Rat brain tissue	Rat brain tissue		Rat brain tissue			
					
Model of ischemia	Permanent focal MCAO	Transient global MCAO	Permanent focal MCAO	Transient focal MCAO	Transient focal MCAO	Permanent focal MCAO	Permanent focal MCAO	Transient focal MCAO	Transient focal MCAO	Blood from ischemic stroke patients	Permanent and transient focal MCAO	Blood from ischemic stroke patients	Post-mortem brain tissue from 11 stroke patients	Post-mortem brain tissue from 12 stroke patients and permanent focal rat MCAO

No of genes	750	374	1176	1263	9044	~8,000	~13,000	5,000	1,322	~19,000	8784	~39,000	7458	1176

Time after ischemia	3 hours	4 hours 24 hours 72 hours	6 hours	6 hours 24 hours	5 hours	24 hours	1 hours 3 hours 6 hours 24 hours	3 hours 6 hours 12 hours 1 days 2 days 4 days	30 min 4 hours 8 hours 24 hours 3 days 7 days	As soon as possible after hospitalization	24 hours	3 hours 5 hours 24 hours	7–10 days (obtained 2–3 days post-mortem)	1 hour-21 days (rat) and 2–37 days (human, obtained by 6 hours post-mortem)

Cut-off values	2.0-fold	1.7-fold	2.0-fold	2.5-fold	1.7-fold	2.0-fold	3.0-fold	2.0-fold	2.0-fold	-	2.0-fold	1.5-fold	-	2.0-fold

Confirmation of results	In situ hybridization, western blotting	Western blotting, immuno-histochemistry	RT-PCR	Real-time PCR, antisense knockdown, western blotting, immuno-histochemistry	Microarray analysis only	Real-time RT-PCR	Cell culture, in situ hybridization, western blotting, immuno-fluorescence	Cell culture, northern blotting, RT-PCR, western blotting, immuno-histochemistry	Real-time RT-PCR	Real-time RT-PCR	Microarray analysis only	Microarray analysis only	Real-time PCR, immuno-histochemistry	Cell culture, RT-PCR, western blotting, immuno-histochemistry, immuno-fluorescence
					
Selected molecules	NGFI-C ARC	GRB2 SMN1	IFN-IP NDGAP-1 NPR	SOCS-3		NARP SPR SPIN2C ARG1 LBP	PC4	FAK	Synaptic proteins	CD14 CD36 FcGR2A IFNGR1 caspase-1 a-catenin			LY64 ELK3 POU3F4 RHOA	PAK1 MMP11 INI1

Until recently, gene expression profiling had not been applied to patients dying of ischemic stroke, in part because human brain autopsies are not regularly obtained. Although tissue obtained from brain autopsies is generally of lower quality than that of brain biopsies obtained from living patients, the majority of RNA transcripts and proteins in the human brain are reasonably stable (compared to other tissues such as blood and kidney) and degrade to only a minor degree following death, thus making autopsy tissue a useful source for the isolation of nucleic acids and proteins [21]. Previous studies evaluating the mRNA quality in human post-mortem brain tissue have demonstrated a minimal effect upon their overall relative stability and indicated that frozen brains up to 72 hours post-mortem can be efficiently analyzed [22]. In line with that, in previous human brain studies, tissue was obtained up to a maximum of 6 hours [7], 40 hours [23], 45 hours [24] and 69 [6] hours following death. Moreover, after comparing mRNA levels in autopsies and biopsies, Castensson et al. [25] found a general similarity in the levels between the two groups, and suggested that mRNA levels in brain autopsy samples can provide clues about the brain in vivo. Interestingly, Almeida et al. [26] found that, even if performed on degraded RNA, RT-PCR can be used to provide a reliable estimate of in vivo mRNA levels, maybe due to the similarities in the rates of degradation between the target and reference mRNAs. Recently, Vikman and Edvinsson [27] investigated the gene expression in human brain after ischaemia using samples 7–10 days post-stroke; however, they obtained their samples after a considerable delay of 2–3 days post-mortem and they focused mainly on mRNA expression of receptors.

To identify the genes whose expression was changed in the human brain following ischaemia, we investigated the dynamic changes in gene expression in brain samples (collected within 6 h of death) from patients with various times of survival (2–37 days; Table 2) following stroke and compared them with those at various time-points (1 hour – 21 days) following middle cerebral artery occlusion (MCAO) in rats. The Atlas 1.2 cDNA microarray was used to screen for differential expression of 1176 genes and significantly de-regulated genes were selected through image analysis. We further investigated whether the altered mRNA and protein levels of a subset of deregulated molecules in the postischemic brain could be reproduced in an *in vitro *model of neuronal and endothelial cell culture under conditions of oxygen-glucose deprivation (OGD). The findings confirmed previous studies reporting that parallel screening of gene expression can detect both previously documented and novel transcriptional features of the cerebral response to ischemia, and demonstrated significant differences in gene expression between human stroke and the animal model.

**Table 2:** Clinical Details of Patients

Patient no.	Age/sex	Survival after stroke	NIHSS on admission	Hypertension^a^	Coronary artery disease	Atrial fibrillation	History of TIA/previous stroke	Hypercholesterolemia^b^	Smoking	Obesity^c^	Cause of death	Antiplatelets	Statins^d^	RSA-b^e^
1	63/F	2 days	26	Yes	No	No	No	No	No	No	Large ischemic stroke	No	No	Yes
2	84/M	3 days	21	Yes	Yes	No	No	No	Yes	No	Malignant stroke	No	No	No
3	68/M	3 days	24	Yes	Yes	No	No	Yes	No	No	Brain oedema	No	No	Yes
4	84/M	6 days	22	Yes	Yes	No	No	No	No	No	Cardiac failure	Yes	Yes	No
5	51/M	9 days	25	Yes	No	No	Yes	No	No	No	Respiratory infection	Yes	No	No
6	74/M	15 days	22	Yes	Yes	No	No	No	No	Yes	Heart attack	Yes	No	Yes
7	86/M	15 days	14	Yes	Yes	No	No	Yes	No	No	Urinary infection	Yes	No	No
8	58/M	17 days	16	Yes	Yes	No	No	Yes	Yes	No	Cardiac infarction	Yes	No	No
9	74/M	20 days	12	Yes	Yes	No	No	No	No	No	Bronchial aspiration	Yes	No	No
10	73/M	26 days	14	Yes	Yes	No	No	Yes	No	Yes	Respiratory infection	Yes	No	Yes
11	75/M	29 days	20	No	Yes	Yes	No	No	Yes	No	Septic shock	Yes	No	Yes
12	60/F	37 days	18	Yes	Yes	No	No	Yes	Yes	No	Pulmonary embolism	Yes	No	No

^a ^Blood pressure greater than 135/85 mmHg.

^b ^Serum total cholesterol levels greater than 5.2 mmol.

^c ^Body mass index greater than 30.

^d ^Patients who were on statins before the ischemic stroke.

^e ^Patients taking either angiotensin converting enzyme inhibitors or angiotensin type I receptor antagonists.

M = male; F = female; NIHSS = NIH Stroke Scale; TIA = Transient Ischaemic Attack.

## Results

### cDNA microarray analysis

**Figure 1:**
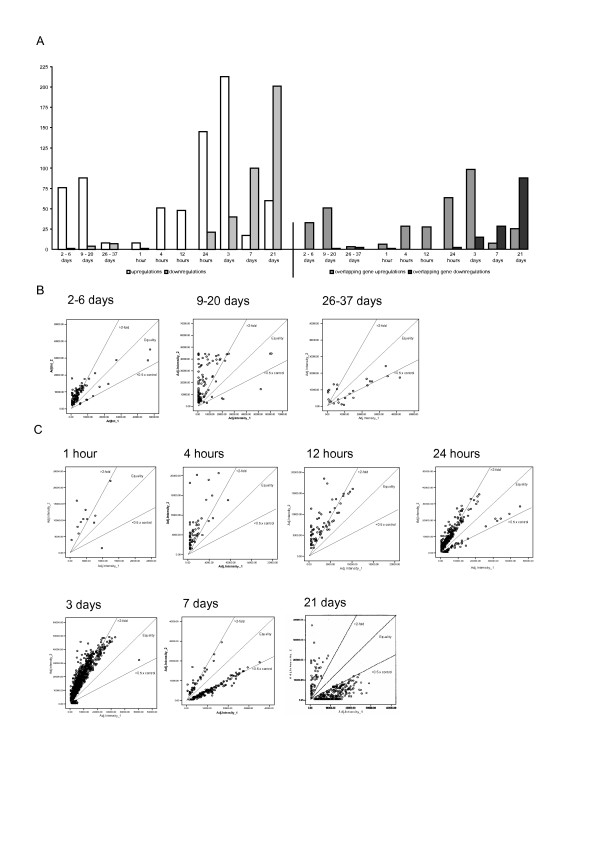
**Statistical analysis of microarray data**. Total number of genes and number of overlapping genes (between the two array sets) deregulated following stroke in human and rat (A). Scatter plots representing the data dispersion over two logarithmic scales for all time-points in human (B) and rat (C).

The expression of ischemia-related genes was determined by comparing the infarct-induced expression (combined samples from infarcted and peri-infarcted areas) to that in the contralateral hemisphere: 77, 92 and 15 genes were de-regulated in stroke-affected regions in the 3 patient survival groups respectively, while 9, 51, 48, 166, 253, 117 and 261 genes were altered at the 7 different time-points in the animal model compared to the controls (Figure 1). The combined number of differentially expressed transcripts in stroke patients represented 6.5%, 7.8% and 1.3% respectively in each survival group of the total number of the genes on the microarray. These findings compare with 0.8%, 4.3%, 4%, 14.1%, 21.5%, 10% and 22.2% of genes respectively at each time-point in rats.

In total, 126 genes were deregulated after stroke in humans and 335 in the rat MCAO model. However, these data are not directly comparable since many transcripts in the human array were not present in the rat array and *vice versa*. Out of a total of 393 genes present in both arrays, 31, 49 and 5 showed deregulated expression in the 3 patient groups respectively, whilst 7, 27, 26, 62, 107, 34 and 107 genes were deregulated at each of the 7 time-points respectively following rat MCAO (Table 3, Figure 1). Of the 393 overlapping transcripts, the expression of 36 was changed only in the human study, compared with 184 that were altered only in the animal model, while only 41 deregulated genes were shared between the two studies. Interestingly, the mean fold changes in the human data were much higher than in the rat.

**Table 3:** Genes deregulated in both human and animal stroke microarrays

	Human		Rat		Human		Rat	
	
Gene name	GenBank	SwissProt	GenBank	SwissProt	Max/min	Days	Max/min	Time
c-jun proto-oncogene	J04111	P05412	X17163	P17325	4.4-fold	9 – 20	3.5-fold	1 h – 24 h
Matrix metalloproteinase 11	X57766	P24347	U46034	P97568	3.2-fold	2 – 20	2.6-fold	3 days
Calcium/calmodulin-dependent kinase (CAMK1)	L41816	Q14012	L24907	Q63450	17.2-fold	2 – 20	0.05-fold	21 days
			L26288	Q63084				
LIM domain kinase 1	D26309	P53667	D31873	P53669	3.6-fold	2 – 20	2.4-fold	3 days
							0.4-fold	21 days
T-Lymphocyte maturation-associated protein	M15800	P21145	U31367	Q64349	1.7-fold	2 – 6	0.2-fold	21 days
Retinoic Acid Receptor beta	M84820	P28702	M81766	P49743	2.0-fold	2 – 6	0.1-fold	21 days
	S54072	P28703						
Tyrosine Phosphatase 1B	M31724	P18031	M33962	P20417	3.4-fold	2 – 6	0.2-fold	21 days
Adenosine A1 Receptor	S56143	P30542	M64299	P25099	2.6-fold	2 – 6	5.2-fold	4 hrs
Growth arrest & DNA damage-inducible 153	S40706	P35638	U30186	Q62804	2.4-fold	2 – 6	2.1-fold	3 days
	S62138							
Glutamate Decarboxylase 67	M81883	Q99259	M34445	P18088	5.6-fold	2 – 6	2.5-fold	21 days
Glutamate Decarboxylase 65	M81882	Q99259	M72422	Q05683	22.7-fold	2 – 20	2.2-fold	3 days
Neurotrophin 3	M37763	P20783	M34643	P18280	5.1-fold	2 – 37	2.2-fold	12 hrs
Inhibitor of DNA binding 2	M97796	Q02363	D10863	P41137	5.6-fold	2 – 20	0.4-fold	21 days
Neuropeptide Y	K01911	P01303	M20373	P07808	8.8-fold	2 – 20	0.04-fold	21 days
Glia Maturation Factor beta	M86492	P17774	Z11558	Q63228	7.6-fold	2 – 6	0.04-fold	21 days
High Mobility Group Protein 1	M23619	P17096	M64986	P27109	4.3-fold	2 – 37	3-fold	4 h – 3 d
				P27428			0.3-fold	21 days
Early Growth Response Protein 1	X52541	P18146	M18416	P08154	4.4-fold	2 – 20	3.9-fold	1 h – 12 h
	M62829		J04154				0.2-fold	21 days
TAT-Binding Protein 1	M34079	P17980	U77918	P97638	3.8-fold	2 – 20	0.4-fold	21 days
Glutathione S-Transferase 1	J03746	P10620	J03752	P08011	17.5-fold	2 – 20	10.8-fold	24 h – 21 d
Fibroblast Growth Factor Receptor 1	M63887	Q02063	D12498	Q04589	10.1-fold	2 – 20	4-fold	4 h – 24 h
	M63888	Q02065						
	M63889							
Interleukin 10	M57627	P22301	L02926	P29456	2.4-fold	2 – 20 26 – 37	6.4-fold	21 days
				Q63263	0.2-fold			
Heat Shock Protein 27	X54079	P04792	M86389	P42930	0.6-fold	2 – 20	15.2-fold	4 h – 24 h
Heat Shock Protein 70	M11717	P08107	Z27118	Q63718	0.6-fold	2 – 6	9.4-fold	1 h – 24 h
		P19790						
Thioredoxin Peroxidase 1	L19185	P32119	U06099	P35704	4.9-fold	2 – 20	3.9-fold	21 days
	X82321	P31945						
Platelet-Derived Growth Factor A	X06374	P04085	L06894	P28576	1.6-fold	2 – 6	0.5-fold	21 days
Matrix Metalloproteinase 14	X83535	Q92678	X83537	Q10739	6.9-fold	2 – 6	3.3-fold	24 h – 3 d
Kinase receptor TYRO3 Sky proto-oncogene	D17517	Q06418	D37880	P55146	3.1-fold	9 – 20	4.0-fold	24 h – 3 d
							0.4-fold	21 days
CSF-1-Receptor	X03663	P07333	X61479	Q00495	89.2-fold	9 – 20	2.8-fold	3 days
Insulin-like Growth Factor Binding Protein 2	M35410	P18065	J04486	P12843	79.7-fold	9 – 20	2.1-fold	3 days
Mitogen activated kinase 1/2	M84489	P28482	M64300	P27703	48.6-fold	9 – 20	0.3-fold	21 days
Aquaporin 4	U34846	P55087	U14007	P47863	18-fold	9 – 20	3.2-fold	3 days
erbB2 proto-oncogene Neu proto-oncogene	M95667	P04626	X03362	P06494	11.2-fold	9 – 20	2.7-fold	12 hrs
	M11730	Q14256						
L-type calcium channel β3	U07139	P54284	M88751	P54287	10.3-fold	9 – 20	8.6-fold	21 days
Ras-related protein RAB3A	M28210	P20336	X06889	P05713	13.9-fold	9 – 20	0.3-fold	21 days
CAMK-II beta	U50358	Q13554	M16112	P08413	1.8-fold	9 – 20	0.3-fold	21 days
Growth Factor Receptor-Bound 2	L29511	Q63057	D49846	Q63057	19.9-fold	9 – 20	2.7-fold	3 days
	M96995	Q14450		Q14450				
Signal Transducer & Activator of Transcription 3	L29277	P40763	X91810	P52631	0.4-fold	9 – 20	6.6-fold	4 h – 3 d
							0.05-fold	21 days
Neuronatin	U25033	Q16517	U08290	Q62649	11.1-fold	9 – 20	0.4-fold	21 days
				Q62663				
Glutathione S-Transferase P	X08058	P09211	X02904	P04906	3.1-fold	9 – 20	0.1-fold	1 hr
Glucocorticoid-regulated serine/threonine kinase GSK	AJ000512	O00141	L01624	Q06226	0.6-fold	26 – 37	2.4-fold	3 days
							0.05-fold	21 days
Glucose Transporter 1	K03195	P11166	M13979	P11167	0.6-fold	26 – 37	11.6-fold	4 h – 21 d

Amongst these genes we examined in more detail a small subset with no prior report of a role in stroke (PAK1, MMP11 and INI1). PAK1 was only induced in the human study although present in both microarray sets, MMP11 was induced in both cases, while INI1 was induced in the human but was not present in the rat microarray set. To confirm the microarray data, RT-PCR was carried out on selected deregulated genes. The temporal expression patterns of these genes following RT-PCR showed good agreement with the corresponding expression profiles obtained from the microarray analysis, supporting the validity of the data obtained from the microarrays. Using Western blotting and immunohistochemistry, PAK1, INI1 and MMP11 protein expression and localization was determined in the contralateral and ipsilateral brain areas of individual stroke patients and rats subjected to MCAO, and in HBMEC and HFN exposed to OGD and reperfusion.

### Integrase Interactor 1 (INI1)

**Figure 2:**
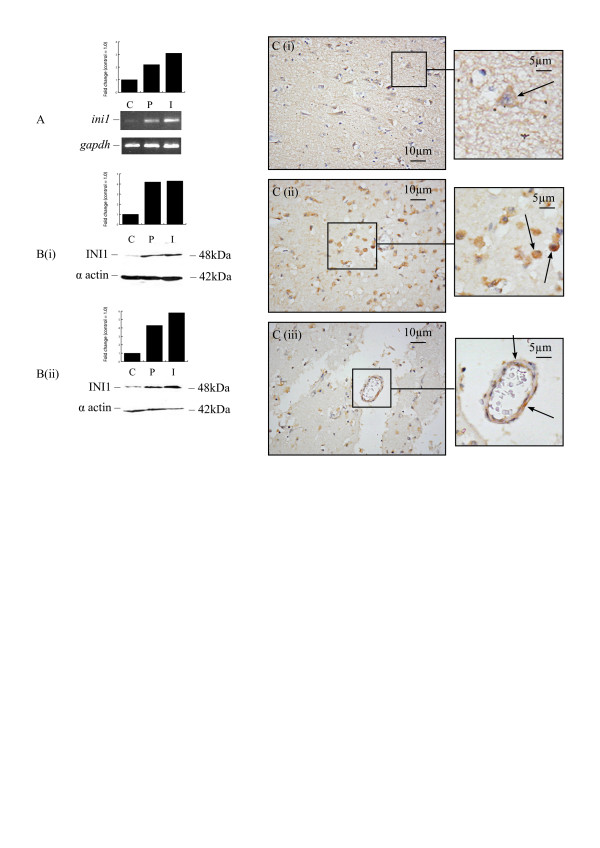
**INI1 expression in human brain following stroke**. RT-PCR demonstrated an increase in *ini1 *mRNA levels in infarcted and peri-infarcted areas of pooled samples from patients surviving from 2 to 6 days following stroke (A). Western blotting showed an increase in protein levels in infarcted and peri-infarcted areas of patients surviving for 3 (Bi) and 6 (Bii) days following stroke. Moderate INI1 neuronal staining (arrow) in contralateral areas of a patient surviving for 3 days after stroke (Ci). Strong INI1 staining in cells (arrows) from infarcted areas of a patient surviving for 15 days after stroke (Cii and iii) (C: Contralateral, P: Peri-infarct, I: Infarct).

In agreement with the microarray data, RT-PCR demonstrated an increase in *ini1 *mRNA levels in peri-infarcted and infarcted areas of patients who survived between 2 and 6 days following stroke (Figure 2A). Analysis of INI1 protein expression in samples from individual stroke patients showed that protein levels were increased in peri-infarcted and infarcted regions in 8 of 12 samples (Table 4; Figure 2Bi and 2Bii). Only one patient who survived for 3 days after stroke showed decreased protein expression. Cells from contralateral white matter were not stained for INI1 but some weak neuronal cytoplasmic staining was seen in grey matter (Figure 2Ci). An increase in its expression was observed in the cytoplasm of cells with the morphological appearance of glia and microvessels from peri-infarcted and infarcted areas of patients surviving for 3 to 29 days after stroke (Figure 2Cii and 2Ciii). In the rat, RT-PCR and Western blotting demonstrated no notable changes in INI1 mRNA and protein expression respectively following MCAO. Weak cytoplasmic staining was observed in contralateral neurons but no differences in the level of INI1 neuronal expression occurred following MCAO (data not included). Finally, HFN and HBMEC exposed to OGD and/or reperfusion showed no difference in mRNA and protein levels for INI1 when compared with untreated cells.

**Table 4:** Protein expression in infarcted (I) and peri-infarcted (P) areas (Fold increase compared to contralateral hemisphere)

		PAK1		INI1		MMP11	
		
Patient no.	Survival (days)	P	I	P	I	P	I
1	2	2.2	1.0	1.5	1.5	1.5	1.5
2	3	3.3	4.0	0.2	0.4	1.0	1.0
3	3	1.0	1.0	4.2	4.3	0.7	0.7
4	6	1.0	1.0	4.3	5.8	1.6	1.5
5	9	1.5	0.4	3.2	3.3	ND	ND
6	15	2.3	1.5	2.8	1.0	1.7	1.6
7	15	3.0	3.2	1.7	2.0	1.0	1.0
8	17	1.0	1.0	1.0	1.0	1.0	1.0
9	20	1.0	1.0	1.0	1.0	1.0	1.0
10	26	1.5	1.5	1.0	1.6	5.1	2.2
11	29	1.0	1.5	2.2	2.8	1.8	3.5
12	37	1.5	1.0	1.7	1.7	1.0	1.5
Total	Upregulated	7	5	8	8	5	6
	Downregulated	0	1	1	1	1	1
	No change	5	6	3	3	5	4
	No detection	0	0	0	0	1	1

### Matrix Metalloproteinase 11 (MMP11)

**Figure 3:**
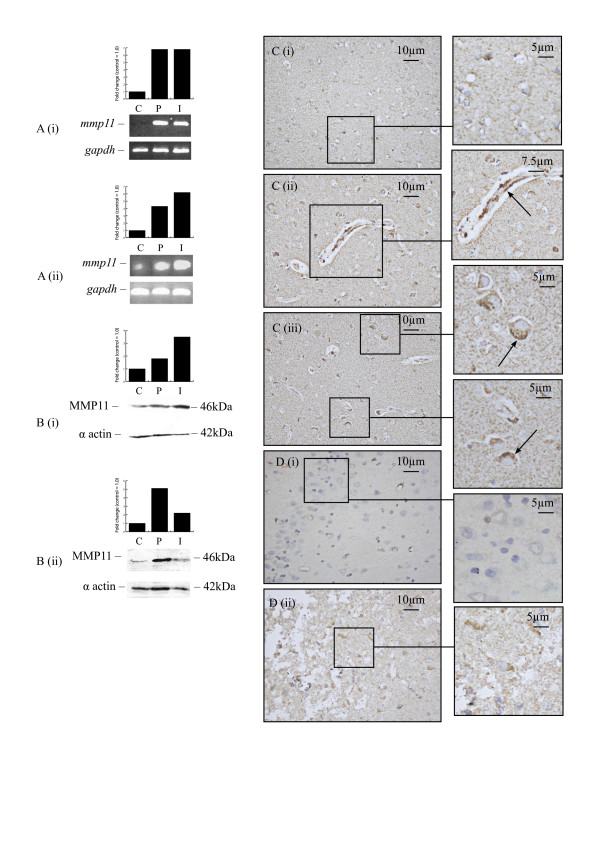
**MMP11 expression in human and rat brain following stroke**. RT-PCR demonstrated an increase in MMP11 mRNA levels in infarcted and peri-infarcted areas of patients surviving from 2 to 6 days following stroke (Ai) and rats at 3 days after MCAO (Aii). Western blotting demonstrated an increase in protein levels in infarcted and peri-infarcted areas of patients surviving for 29 (Bi) and 26 (Bii) days following stroke. Weak MMP11 staining in cells from contralateral areas of a patient surviving for 5 days following stroke (Ci). Blood vessels (Cii) and neurons (Ciii) strongly stained for MMP11 in peri-infarcted areas of a patient surviving for 15 days after stroke (arrows). No MMP11 staining observed in contralateral hemisphere of rat brain at 1 h after MCAO (Di) but neurons from infarcted areas of rat brain were stained positive for MMP11 at 3 days following MCAO (Dii) (C: Contralateral, P: Peri-infarct, I: Infarct).

For MMP11, RT-PCR data agreed with the findings from the microarray study, showing increased mRNA levels in infarcted and peri-infarcted tissue from patients surviving 2–20 days following stroke (Figure 3Ai). Western blotting in individual patient samples demonstrated that 6 of 12 patients had elevated MMP11 protein levels (Table 4; Figure 3Bi and 3Bii). The majority of cells from contralateral grey and white matter were not stained for MMP11 (Figure 3Ci). In patients surviving from 3 days to 4 weeks, endothelial cells and neurons from both infarcted and peri-infarcted tissue were stained positive for MMP11 (Figure 3Cii and 3Ciii). In the rat model, RT-PCR confirmed the microarray data for some of the time-points, showing no notable change in mRNA levels at 1 and 12 h but a prolonged upregulation at 3 days following MCAO (Figure 3Aii). Protein levels were elevated at 1 h, 24 h and 3 days, after which they returned to control levels. No staining for MMP11 was seen in contralateral areas (Figure 3Di), but an increase in its expression occurred in neurons following MCAO, in particular at 12 and 24 h (Figure 3Dii). MMP11 mRNA and protein levels remained unchanged in HFN and HBMEC exposed to conditions of oxygen-glucose deprivation.

### P21-activated kinase 1 (PAK1)

**Figure 4:**
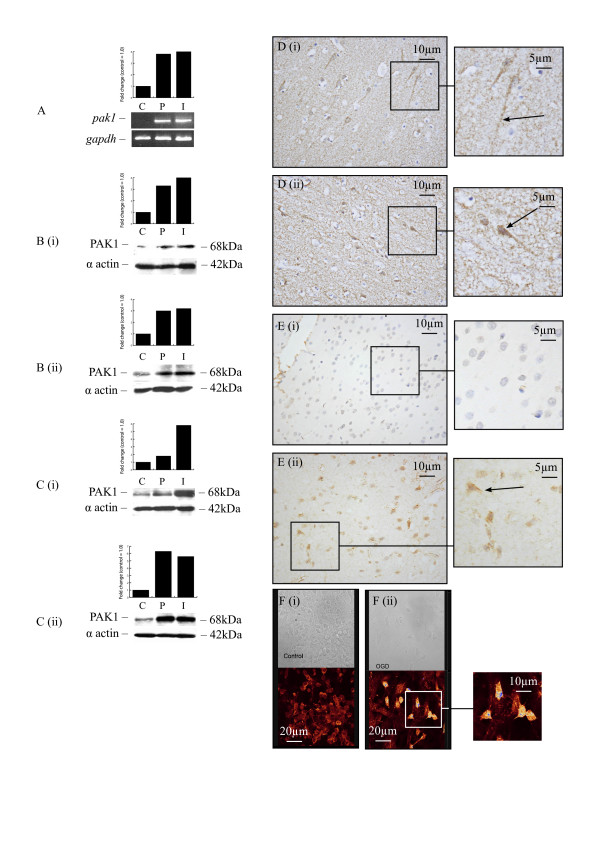
**PAK1 expression in human and rat brain following stroke**. RT-PCR demonstrated an increase in PAK1 mRNA in infarcted and peri-infarcted areas of pooled samples from patients surviving from 2 to 6 days following stroke (A). Western blotting demonstrated an increase in protein levels in infarcted and peri-infarcted areas of patients surviving for 3 (Bi) and 15 (Bii) days following stroke and in rats at 12 h (Ci) and 24 h (Cii) following MCAO. Weak neuronal (axonal) staining (arrow) observed in contralateral areas of a patient surviving for 15 days following stroke (Di). Strong PAK1 staining in neurons (arrow) and cells with the morphological appearance of glia from infarcted areas of a patient surviving for 3 days following stroke (Dii). No staining observed in contralateral areas of rat brain at 24 h following MCAO (Ei) while strong PAK1 staining was seen in neurons (arrow) and cells with the morphological appearance of glia from infarcted areas of rat brain 1 h following MCAO (Eii). Stronger PAK1 immunofluorescent staining was seen in HFN following OGD (Fii) compared to control (Fi) (C: Contralateral, P: Peri-infarct, I: Infarct).

RT-PCR confirmed the upregulation of *pak1 *determined by the microarrays in pooled samples from stroke patients who survived between 2 and 6 days following stroke (Figure 4A). Western blotting showed an upregulation in the protein levels of PAK1 in 6 of 12 patients (Table 4; Figure 4Bi and 4Bii). No staining was seen in contralateral white matter, while, in grey matter, PAK1 stained weakly the cytoplasm of some neurons (Figure 4Di). In patients surviving for 3 days to 4 weeks after stroke, increased PAK1 nuclear staining was seen in neurons in both peri-infarcted and infarcted regions (Figure 4Dii). In the rat, RT-PCR showed no significant changes in the mRNA levels for *pak1 *at most of the time-points examined. However, Western blotting showed an upregulation in protein levels 1, 12 and 24 h after MCAO, returning to control levels at 3 days, and becoming downregulated at 7 days following MCAO (Figure 4Ci and 4Cii). Weak staining was observed in neurons from the contralateral hemisphere (Figure 4Ei), but an increase in cytoplasmic and nuclear staining in neurons occurred following MCAO, in particular at 1 h and 24 h (Figure 4Eii). Finally, an increase in PAK1 expression was also seen in human foetal neurons following oxygen-glucose deprivation (Figure 4Fi and 4Fii).

## Discussion and Conclusion

In the human brain, many differentially expressed genes were observed from 2 to 6 days and from 9 to 20 days after stroke, with the majority being upregulated. The number of deregulated genes declined during 26 to 37 days after stroke, indicating that dynamic changes in gene expression occur during the first days to few weeks in the human postischaemic brain. In the rat brain, few differences were observed at 1 hour, while the number of differentially expressed genes steadily increased with time after MCAO, with a peak after 3 days, supporting the concept of active mechanisms initiated during the acute phase after experimental stroke and lasting for several days. The number of upregulated genes gradually increased, peaking at 3 days, while downregulated genes were detected 24 h after MCAO and increased dramatically until the final measured time-point at 21 days (Figure 1).

The limitations of post-mortem brain samples in cDNA microarray analysis concern the small sample size and potential low quality and the genetic heterogeneity and diversity in terms of age, sex and previous medical history within a group of patients [28,29]. We found that analysis of postischaemic gene expression using a cDNA microarray can allow identification of known and novel transcriptional events, molecular participants and signalling mechanisms in cerebral ischaemia as previously suggested, but can also detect differences in gene expression between distinct organisms.

The present gene expression profile study is the first large-scale microarray report showing altered expression of several genes following human stroke. These included genes participating in transcription, apoptosis, inflammation and neuroprotection. Many genes/proteins previously shown to be deregulated following stroke were reported in our study too e.g. IL-10 [30,31], PDGF [32], STAT3 [33,34], MAPK1/2 [35]. To test whether our microarray analysis could predict novel candidate genes involved in the cerebral response to ischaemia with possible functional importance and significance in stroke-induced neuronal damage, we measured protein expression and cellular localisation for three induced genes, INI1, PAK1 and MMP11. They were chosen because they showed at least 2-fold mRNA induction and there was no prior published evidence implicating them in human cerebral ischaemia.

PAK1 is a downstream Rac effector and a major cyclin-dependent kinase 5 (Cdk5) substrate and target that co-localizes with p35/Cdk5 at neuronal peripheries. P35/Cdk5 causes PAK1 hyperphosphorylation, which results in PAK1 down-regulation and is likely to have an impact on the dynamics of the reorganization of the actin cytoskeleton in neurons during dendrite development [36]. Based on this evidence, these authors proposed the existence of a neuron-specific signalling complex involving Cdk5/p35-PAK1 that inhibits PAK1 activity in neurons. We have recently provided evidence for a potential role of Cdk5/p35 in the response to ischaemic injury as we showed association of Cdk5 with nuclear damage, by demonstrating co-expression of Cdk5 in TUNEL-positive neurons following human stroke and in propidium iodide-positive human foetal neurons following OGD [37]. Here, we have reported for the first time an upregulation in PAK1 protein levels in human and rat brain samples following MCAO and in HFN following oxygen-glucose deprivation. Although in the animal model PAK1 protein levels returned to normal 3 days following stroke, some patients showed elevated levels for PAK1 at later time-points too. In both human and the animal model, neurons were the predominant type of cells stained positive for PAK1.

MMP11 or stromelysin-3 (ST3), first isolated as a breast cancer-associated protease, is not expressed in the majority of normal adult organs but is expressed during a number of pathological processes, including wound healing and atherosclerotic lesions [38,39]. Although other metalloproteinases have been studied extensively following stroke [40,41], there is no report of the expression of MMP11 following stroke *in vivo *or *in vitro*. Here we report an increase in protein levels of MMP11 following stroke in both human and rat brain, although the increase seen in man remained elevated much longer. Although MMP11 shares many similarities with other MMPs, it also differs in that it exhibits anti-apoptotic properties, a first-known activity for a MMP [42]. Moreover, although it is expressed in many processes involving tissue remodelling, cell migration and cell death, the pathways through which it participates in pathogenesis remain unclear, largely due to the lack of information on its substrates *in vivo *[43].

INI1 is a tumour suppressor gene, thought to exert its tumour suppressor function by mediating cell cycle arrest [44]. It was initially identified as a human homolog of yeast transcriptional activator SNF5 that binds to the HIV-1 integrase and stimulates its DNA-joining activity [45]. Brains of AIDS patients had been shown to manifest neuronal injury and apoptotic-like cell death raising the question about the way HIV-1 resulted in neuronal damage, since neurons themselves are very rarely infected by the virus [46]. Adler *et al*. [47] also reported an association of the human SNF5/INI1 protein with growth arrest and DNA damage-inducible protein 34 (GADD34) that mediates growth arrest and apoptosis in response to stress signals [48,49]. Our study is the first to suggest a potential role for INI1 in pathways activated after stroke with a possible role in brain injury. However, in the animal model study, INI1 levels remained unchanged following stroke. The reason for this discrepancy warrants further studies.

Many experimental trials of stroke therapies have failed to translate to human clinical trials and one possible way to improve the success rate can be through comparative genomics. As it has been recently commented, it is very surprising that the exciting developments observed in basic and clinical stroke research over the past two decades have occurred in parallel, with too little direct translation between bench and bedside [50]. Here, we have provided substantial evidence that, although the available animal models of MCAO may well be suitable to study the pathophysiological changes following the occlusion of a cerebral vessel, they may not entirely reflect the pathophysiological process through which stroke evolves in humans. The species difference is one of the main reasons accounting for the lack of success of bench to bedside translation in the stroke area. Limitations of our study include the fact that early acute phase changes in gene expression may have been missed since genes induced and returning to normal during the first 48 hours post-ischaemia in man could not have been detected. Moreover, since we analyzed pooled RNA samples, small changes in gene expression occurring in a minority of the samples may have been missed. However, there was only a small overlap of our results with prior studies in experimental stroke involving brain tissue, and the successful identification of novel ischaemia-related genes reported here suggests that performing a further study using whole genome microarrays would be valuable.

## Methods

### Human brain autopsy specimens

Human brain tissue samples were obtained from 12 patients who died from acute ischaemic stroke, with the approval of the local Ethics Committee and Brain Bank at the Department of Neuropathology, Collegium Medicum, Jagiellonian University, Krakow, Poland. All patients were admitted with large middle cerebral artery strokes confirmed by CT-scan or MRI. The patients, 10 male and 2 female, were aged between 51 and 86 years and had survived between 2–37 days following ischaemic stroke (Table 2). Routine blood parameters were determined on admission. Full clinical examinations, including NIH Stroke Scale, were also carried out on admission. Excluded from the study were patients with recent history of head trauma, major cardiac, renal, hepatic or cancerous disease and obvious signs of infection after admission. Immediately after death the body was put in a cold chamber and tissue was collected within 6 h of death. Tissue samples were taken from infarct and peri-infarcted zones while controls were obtained from the contralateral hemisphere at the same time. The peri-infarcted areas were defined in tissue sections as the tissue immediately surrounding the infarcted core which contained some necrotic cells and showed evidence of tissue disorganisation confirmed by histology. Sections were stained with 2,3,5-triphenyltetrazolium chloride which stains active mitochondria pink; therefore, non stained areas represented stroke affected cortical regions (data not included). Tissue specimens were immediately frozen in liquid nitrogen, kept at -70°C and a portion of each sample was processed for histology and stained with haematoxylin and eosin to determine tissue morphology [51].

### Rat middle cerebral artery occlusion

Stroke experiments were performed on female Sprague-Dawley rats (weight: 230–270 g) as they suffer less than male during ischaemia. Cerebral ischaemia was produced using a modified method of Baron [52] by distal, permanent occlusion of the MCA by electrocautery as described elsewhere [53,54]. The mortality in this model is very low. Sets of six animals (3 for morphological studies and 3 pooled together) for each time-point were sacrificed at 1 h, 4 h, 12 h, 24 h and 3, 7 and 21 days.

### In vitro oxygen-glucose deprivation (OGD)

Human brain microvascular endothelial cells (HBMEC) were obtained from TCS CellWorks (Buckingham, UK) and cultured according to the supplier's instructions. Human foetal (cerebral cortical) neurons (HFN) were extracted and cultured with permission from the Local Ethics Committee. Brain tissue from foetus specimens of 14–19 weeks gestational age, legally aborted and with the appropriate written consent, were collected in cold preservation medium and cells were isolated and cultured as described elsewhere [55]. For OGD experiments, the culture medium was replaced by glucose-free medium containing 2% foetal bovine serum (TCS CellWorks, Buckingham, UK) and cells were cultured at 37°C in a humidified chamber with 94% N_2_, 1% O_2_, and 5% CO_2 _for 6 h (HBMEC) or 95% N_2 _and 5% CO_2 _for 14 h (HFN) followed by 24 h reperfusion in fresh medium containing 4.5 g/l glucose. This resulted in approximately 30% of cells undergoing apoptosis after OGD and 60% following reoxygenation, as determined from our pilot studies. Cells cultured in normoxic conditions without glucose deprivation were used as controls. In some experiments, propidium iodide (10 μg/ml) was added to the cultures 1 h before the end of the experiment to stain dead and dying cells.

### cDNA microarrays

We established mRNA expression profiles of the damaged brain tissues between 2 to 6 days, 9 to 20 days, and 26 to 37 days after stroke in human patients and 1, 4, 12, 24 hours and 3, 7 and 21 days after the ischaemic insult in rats. The corresponding samples from the non-ischemic control hemisphere were used to measure the normal mRNA abundance of the modulated genes in each tissue at each time point. RNA from three stroke patients was pooled for each patient survival group while RNA from three MCAO rats was also pooled at each time-point to improve yields in preparation of poly A^+ ^RNA. Although pooling was previously thought to affect data quality, Kendziorski *et al. *[56] have recently shown that inference was not adversely affected by pooling. The different patient groups were selected to match the three physiological stages following stroke i.e. the inflammatory (lasting up to a maximum of 5–6 days), the proliferative (lasting up to three weeks following stroke) and the remodelling/maturation (starting during the third or fourth week).

RNA was extracted according to the manufacturer's protocols (BD Biosciences, Oxfordshire, UK) and its quality was measured spectrophotometically. The protocol recommended by Clontech in their Atlas 1.2 microarray kit was used without any modification. Briefly, RNA was reverse-transcribed to cDNA, ^32^P-labelled and applied to the array for overnight hybridisation at 68°C. Following washing, the array was exposed to a phosphorimaging plate for 12–72 hours and data analysis was performed using the AtlasImage 1.5 software. The results were normalized using two housekeeping genes, ubiquitin and glyceraldehyde 3-phosphate dehydrogenase (GAPDH). As in the majority of microarray studies mentioned before, only those genes upregulated > 2-fold or downregulated < 0.5-fold were counted as deregulated and taken into consideration. The microarray data are available in Gene Expression Omnibus under the accession number GSE9391.

### Reverse Transcription-Polymerase Chain Reaction (RT-PCR)

Gene expression was examined by semi-quantitative RT-PCR with standard reaction conditions of a 10 min denaturation at 94°C, followed by 35 cycles of 1 min at 94°C, 1 min at primer-specific annealing temperatures (Table 5) and 1 min at 72°C and a final 10 min extension step at 72°C. Samples without cDNA were used as negative controls and the products were visualized by agarose gel electrophoresis (1.5% w/v) and DNA stained with ethidium bromide (10 mg/ml). All experiments were carried out at 25, 30, 35 and 40 cycles to ensure the semi-quantitative nature of the results. The results were normalized using housekeeping gene GAPDH and semi-quantitavely analyzed using Scion Imaging Software version 4.02 (Scion Corporation, Maryland, USA). Sense and antisense oligonucleotide primers containing 18–27 nucleotides based on previously reported mRNA sequences in the GenBank depository were designed with the aid of the Primer3 Output Program (Version 0.2). InVitrogen plc. (Paisley, UK) synthesized the primer sets (Table 5).

**Table 5:** Primer sequences

**Gene**	**Species**	**Primer Sequence**	**T annealing**
*mmp11*	Human	5'-TAAAGGTATGGAGCGATGTGAC-3' (forward)	58°C
*mmp11*		5'-TGGGTAGCGAAAGGTGTAGAAG-3' (reverse)	
*mmp11*	Rat	5'-GATGGAGGCCAGCTAGTCAG-3' (forward)	60°C
*mmp11*		5'-ATGGTACATGACCACGCAGA-3' (reverse)	
*ini1*	Human	5'-ACCCTGTCCAACAGCTCCCA-3' (forward)	64°C
*ini1*		5'-GGCCCAATCTTCTGAGATGC-3' (reverse)	
*ini1*	Rat	5'-CCTGGGGCTCCTATACAAAA-3' (forward)	60°C
*ini1*		5'-CCATGACCGAGCAAATGAC-3' (reverse)	
*pak1*	Human	5'-GCTGTTCTGGATGTGTTGGA-3' (forward)	60°C
*pak1*		5'-TCTGCTCTGGGGTTATCTGTG-3' (reverse)	
*pak1*	Rat	5'-AGCAAAAGAGGCAACCAAGA-3' (forward)	60°C
*pak1*		5'-GGGTAAGGAATGGGATGGTT-3' (reverse)	
*gapdh*	Human	5'-ATGATCTTGAGGCTGTTG-3' (forward)	58°C
*gapdh*		5'-CTCAGACACCATGGGGAA-3' (reverse)	

### Protein extraction and Western blotting

Proteins were extracted from tissues and the protein concentration of each sample was determined using the BioRad assay. For Western blotting, 10 μg of protein were separated by SDS-PAGE (13% w/v) and the proteins were electro-blotted onto nitrocellulose filters as described previously [57]. Filters were blocked in 1% w/v bovine serum albumin (BSA) in Tris-buffered saline Tween (TBS Tween) and stained overnight at 4°C with antibodies to the following proteins (obtained from Autogen Bioclear, Wiltshire, UK, unless stated otherwise) diluted in 1% BSA: MMP11 (CalBiochem; 1:500), PAK1 (1:500), INI1 (1:500), and α-actin (Sigma, 1:1000) used as a loading control. Membranes were washed in TBS-Tween before staining with the appropriate peroxidase-conjugated secondary antibody, diluted 1:1000 in 5% w/v milk in TBS-Tween for l h. Blots were developed with the ECL detection system (Amersham, UK). The relative intensities of the bands were measured in an LKB densitometer. Results are semi-quantitative and are given as a numerical (fold) change compared to the control (contralateral tissue) which was given an arbitrary value of 1.0. All experiments were performed twice and a representative example of patient(s) showing an increase in protein expression is given.

### Immunohistochemistry/Immunofluorescence

Paraffin-embedded tissue samples were processed and serial 5 μm sections were cut. The Avidin-Biotin Peroxidase (ABC Vectastain kit, Vector Laboratories, Peterborough, UK) method was used and antibodies to MMP11, PAK1 and INI1 were used at a dilution of 1:50. Paraffin-embedded sections were deparaffinized, rehydrated and boiled for 10 min in an antigen unmasking solution of concentrated citric acid pH 6.0 as described elsewhere [57]. Slides were incubated in 0.5% v/v H_2_O_2 _in methanol for 30 min, with normal serum for 20 min and then with a primary antibody (diluted in normal serum) for 30 min, followed by 30-min incubation with biotinylated secondary antibody (diluted 1:50) and finally with ABC complex (diluted 1:50) for 30 min at RT. Staining was completed after incubation with DAB substrate chromogen solution for 3–10 min. Slides were counterstained with haematoxylin, dehydrated, cleared and mounted in DPX. For immunofluorescence, cultured cells were fixed in 4% paraformaldehyde for 20 min, permeabilized with 0.2% Triton ×100 for 10 min, blocked with normal serum and stained with the primary antibody as above, followed by 1 h incubation with Alexa-fluor conjugated dye at RT. Negative control slides were performed in parallel, where primary antibody was replaced with washing buffer and processed as above (data not included).

## Abbreviations

GAPDH: glyceraldehyde 3-phosphate dehydrogenase; HBMEC: human brain microvascular endothelial cells; HFN: human foetal neurons; INI1: integrase interactor 1; MCAO: middle cerebral artery occlusion; MMP11: matrix metalloproteinase; OGD: oxygen-glucose deprivation; PAK1: p21-activated kinase 1.

## Authors' contributions

NM carried out the human studies and drafted the manuscript. MOS carried out the experimental work in rats. JK and FR provided the material for the study. RP and CS carried out the *in vitro *work. QW participated in the analysis of the microarray results. MS performed the statistical analysis. JG, PK and SK participated in the design and coordination of the study and helped to draft the manuscript. MAS conceived of the study, and directed the research project. All authors read and approved the final manuscript.
